# Cardiac myosin filaments are directly regulated by calcium

**DOI:** 10.1085/jgp.202213213

**Published:** 2022-11-01

**Authors:** Weikang Ma, Suman Nag, Henry Gong, Lin Qi, Thomas C. Irving

**Affiliations:** 1 BioCAT, Department of Biology, Illinois Institute of Technology, Chicago, IL; 2 Department of Biochemistry, Bristol Myers Squibb, Brisbane, CA

## Abstract

Classically, striated muscle contraction is initiated by calcium (Ca^2+^)-dependent structural changes in regulatory proteins on actin-containing thin filaments, which allow the binding of myosin motors to generate force. Additionally, dynamic switching between resting *off* and active *on* myosin states has been shown to regulate muscle contractility, a recently validated mechanism by novel myosin-targeted therapeutics. The molecular nature of this switching, however, is not understood. Here, using a combination of small-angle x-ray fiber diffraction and biochemical assays with reconstituted systems, we show that cardiac thick filaments are directly Ca^2+^-regulated. We find that Ca^2+^ induces a structural transition of myosin heads from ordered *off* states close to the thick filament to disordered *on* states closer to the thin filaments. Biochemical assays show a Ca^2+^-induced transition from an inactive super-relaxed (SRX) state(s) to an active disordered-relaxed (DRX) state(s) in synthetic thick filaments. We show that these transitions are an intrinsic property of cardiac myosin only when assembled into thick filaments and provide a fresh perspective on nature’s two orthogonal mechanisms to regulate muscle contraction through the thin and the thick filaments.

## Teaser

Calcium induces a structural transition of myosin heads from ordered *off* states close to the thick filament to disordered *on* states near the thin filaments associated with a biochemical shift from the super-relaxed state(s) to the disordered-relaxed state(s) of myosin.

## Introduction

Calcium (Ca^2+^) signaling coordinates many different intracellular processes in plant, animal, and human physiology (Bagur and Hajnoczky, 2017). Muscle contraction is one of those biological processes regulated by Ca^2+^ and is propelled by the sliding of actin-containing thin filaments along myosin-containing thick filaments in the sarcomere (Hanson and Huxley, 1953). For over 50 yrs, it has generally been accepted that Ca^2+^ binds to the troponin complex on the thin filament to initiate and regulate this process (Tobacman, 1996). Briefly, under resting conditions, the myosin-binding sites on the thin filament are blocked by tropomyosin, thus preventing muscle contraction. Upon receiving the contraction signals, Ca^2+^ entering the cytosol binds to troponin-C on the thin filament, triggering a series of conformational changes that ultimately unblock the myosin-binding site on actin by physically moving the tropomyosin. Initial binding of myosin to such unblocked sites further leads to the full activation of the thin filament (Lehman et al., 1994; Risi et al., 2021).

However, this textbook Ca^2+^-mediated thin filament-based mechanism does not address how thick filaments are turned on and off. Under relaxed conditions, myosin heads inside the sarcomere may be found in two biochemical states: one with a relatively high ATPase (∼0.03 s^−1^) called the disordered-relaxed (DRX) state(s), and the other, a low ATPase (∼0.003 s^−1^) state known as the super-relaxed (SRX) state(s) (Stewart et al., 2010). X-ray diffraction experiments (Anderson et al., 2018; Yuan et al., 2022) have shown that myosin heads can adopt ordered configurations close to the thick filament backbone where the heads cannot interact with actin and are considered to be in an *off* state(s). Under other conditions, disordered heads are located away from the thick filament backbone where they are free to interact with actin, considered to be in an *on* state(s). The structural *off*-to-*on* transition is thought to closely correlate with the biochemical SRX to DRX transitions of myosin (Craig and Padron, 2022), but it is not yet clear whether these are strictly equivalent. Be that as it may, dynamic switching between the SRX states and the DRX states of myosin has been shown to regulate muscle contractility (Spudich, 2019; Nag and Trivedi, 2021) and has emerged as one of the underlying causes of hyper- or hypo-contractility in myopathies. Understanding the details of thick filament regulation mechanisms, therefore, has implications for understanding the etiology of many myopathies and so aid the development of rational treatment strategies (Alsulami and Marston, 2020).

Different muscle systems have developed different thick filament regulation mechanisms to fulfill their functions. Muscle myosins from invertebrates such as scallops can be activated by direct Ca^2+^ binding to the essential light chain (ELC; Szent-Gyorgyi, 2007), thus actively cycling cross-bridges. Tarantula skeletal muscle and vertebrate smooth muscle activate their thick filaments through Ca^2+^ binding to calmodulin, which activates myosin light chain kinase (MLCK), which in turn phosphorylates the myosin’s regulatory light chain (RLC; Walsh, 1994; Padron et al., 2020) thus turn the thick filament on to participate in force generation. Linari et al. (2015) proposed a strain-dependent thick filament activation model (“mechano-sensing”) for vertebrate skeletal muscle and later expanded this mechanism to rodent cardiac muscle (Reconditi et al., 2017; Caremani et al., 2019). In this mechano-sensing model, when Ca^2+^ is released to the cytoplasm and binds to troponin, thereby activating the thin filament, a small number of myosin heads that are constitutively on can form cross-bridges and generate sufficient force to induce strain in the thick filament. The thick filament acts as a mechanical sensor where the force exerted on the thick filament backbone induces a “structural transition” that releases myosin heads from an inactive *off* state(s) to an active *on* state(s) that increases the proportion of myosin heads competent to bind actin and generate force (Linari et al., 2015; Caremani et al., 2019). It has been shown, however, that passive stretch also can strain the thick filament backbone to a similar degree as with active force, but this strain alone cannot account for the full number of myosin heads that transition from the *off*-to-*on* states seen in actively contracting muscle (Ma et al., 2018b; Ma et al., 2019; Park-Holohan et al., 2021). This suggests that thick filament strain cannot be the only trigger for *off*-to-*on* structural transitions that result in the release of myosin heads from the thick filament backbone. One unexplored candidate for this additional trigger in contracting muscle would be Ca^2+^, the primary difference between active and resting muscle.

Here, using a combination of biochemical (SRX/DRX and ATPase) assays on different myosin constructs and in synthetic thick filaments and small-angle x-ray fiber diffraction on permeabilized porcine myocardium where actin-myosin interaction is prevented using a small molecule inhibitor, we show that cardiac thick filaments are directly Ca^2+^-regulated. This Ca^2+^-mediated thick filament sensing mechanism may have broad implications for understanding the structural basis of calcium regulation of muscle.

## Materials and methods

### Muscle sample preparation

Frozen wild-type left ventricular wall myocardium was provided by Exemplar Genetics LLC. Humane euthanasia and tissue collection procedures were approved by the Institutional Animal Care and Use Committees at Exemplar Genetics LLC. Permeabilized tissues are prepared as described previously (Ma et al., 2021; Ma et al., 2022). Briefly, frozen wild type porcine (*n* = 2) left ventricle wall (about 1 cm^3^) is defrosted in skinning solution (91 mM K^+^-propionate, 3.5 mM MgCl_2_, 0.16 mM CaCl_2_, 7 mM EGTA, 2.5 mM Na_2_ATP, 15 mM creatine phosphate, 20 mM imidazole, 30 mM 2,3-butanedione monoxime, 1% Triton-X100, and 3% dextran at pH 7) at room temperature before dissecting into smaller strips (∼1 cm long and 2–3 mm wide). The tissues are permeabilized at room temperature for 3 h. The tissues are then washed three times, 10 min each, in pCa 8 solution (91 mM K^+^-propionate, 3.5 mM MgCl_2_, 0.16 mM CaCl_2_, 7 mM EGTA, 2.5 mM Na_2_ATP, 15 mM creatine phosphate, 20 mM imidazole, and 3% dextran at pH 7). Well-aligned tissues are further dissected into preparations of 4 mm length and a diameter of ∼200 µm before attaching aluminum T-clips to both ends.

### Cardiac protein purification

Bovine cardiac actin, tropomyosin, and troponin complex are purified following modified methods previously published (Spudich and Watt, 1971). Actin is stored at −80°C as G-actin and polymerized fresh for each day of experiments by adding 50 mM KCl and 2 mM MgCl_2_ to the actin-containing buffer. The regulated thin filament (RTF) system is reconstituted using bovine cardiac actin:bovine cardiac tropomyosin:bovine cardiac troponin complex (1:1:1 troponin-C:troponin-T:troponin-I) in a 7:1:1 ratio. β-cardiac full-length myosin from the bovine left ventricle is isolated following established methods described elsewhere (Margossian and Lowey, 1982). Following this, proteins are dialyzed in a buffer containing 10 mM PIPES (pH 6.8), 300 mM KCl, 0.5 mM MgCl_2_, 0.5 mM EGTA, 1 mM NaHCO_3_, and 1 mM DTT and stored at −80°C. Based on densitometry analysis of SDS-PAGE, the purity of the myosin preparation varies between 90 and 95%, with negligible actin contamination. Also, the basal myosin ATPase from these preps always turns out to be 0.03 ± 0.01 s^−1^, suggesting negligible actin-activation.

Using bovine cardiac full-length myosin as the starting material, heavy meromyosin (HMM) and S1 subfragment are prepared according to methods described in a previous report (Margossian and Lowey, 1982).

Human β-cardiac 2-hep and 25-hep HMM are purified using methods described elsewhere (Nag et al., 2017). These HMM cDNA constructs consist of a truncated version of MYH7 (residues 1–855), corresponding to S1-subfragment and the first 2 heptads (14 amino acids) or 25 heptad repeats (175 amino acids) of S2-subfragment for the 2-hep and 25-hep HMM, respectively, followed by a GCN4 leucine zipper to ensure dimerization. This is further linked to a flexible GSG (Gly-Ser-Gly) linker, then a GFP moiety followed by another GSG linker, and finally ending with an eight-residue (RGSIDTWV) PDZ binding peptide.

### Myosin ATPase measurements from myosin synthetic myosin thick filaments reconstituted from full-length myosin

Methodologies involving the reconstitution of myosin synthetic thick filaments (STF) have been described previously (Gollapudi et al., 2021). Briefly, full-length bovine β-cardiac myosin remains fully soluble in a buffer of high ionic strength (300 mM) but spontaneously self-assembles into bipolar thick filaments at lower ionic strengths (Gollapudi et al., 2021). For each experiment, 10 µM of full-length myosin in 300 mM KCl buffer was diluted to 1 µM in KCl buffer containing 20 mM Tris-HCl (pH 7.4), 0 mM KCl, 1 mM EGTA, 3 mM MgCl_2_, and 1 mM DTT, to achieve a final KCL concentration of 30 mM. To allow for thick filament formation, the sample was incubated for 2 h on ice, and then used in the experiments. Basal STF ATPase activity measurements are performed at 23°C on a plate-based reader (SpectraMax 96-well) using an enzymatically coupled assay as described earlier (Kawas et al., 2017). The buffer composition used for these experiments is 12 mM PIPES (pH 6.8), 2 mM MgCl_2_, 10 mM KCl, and 1 mM DTT. Assuming negligible contribution from the PIPES buffer system, the ionic strength of this buffer is ∼16 mM.

### Use and characterization of MYK-7660

The small molecule, MYK-7660, is a regulated thin-filament-activated myosin ATPase inhibitor. It was discovered initially from a high-throughput compound library screen at MyoKardia Inc., a wholly owned subsidiary of Bristol Myers Squibb. This article uses this small molecule as a tool compound to shut off the regulated thin filaments in the presence of high Ca^2+^. For all experiments, a 20 mM stock in 100% DMSO is used to attain the desired concentration in the final buffer of the experiment with a final 2% DMSO concentration. All control experiments without MYK-7660 are performed in a buffer containing 2% DMSO.

**Figure 1.**
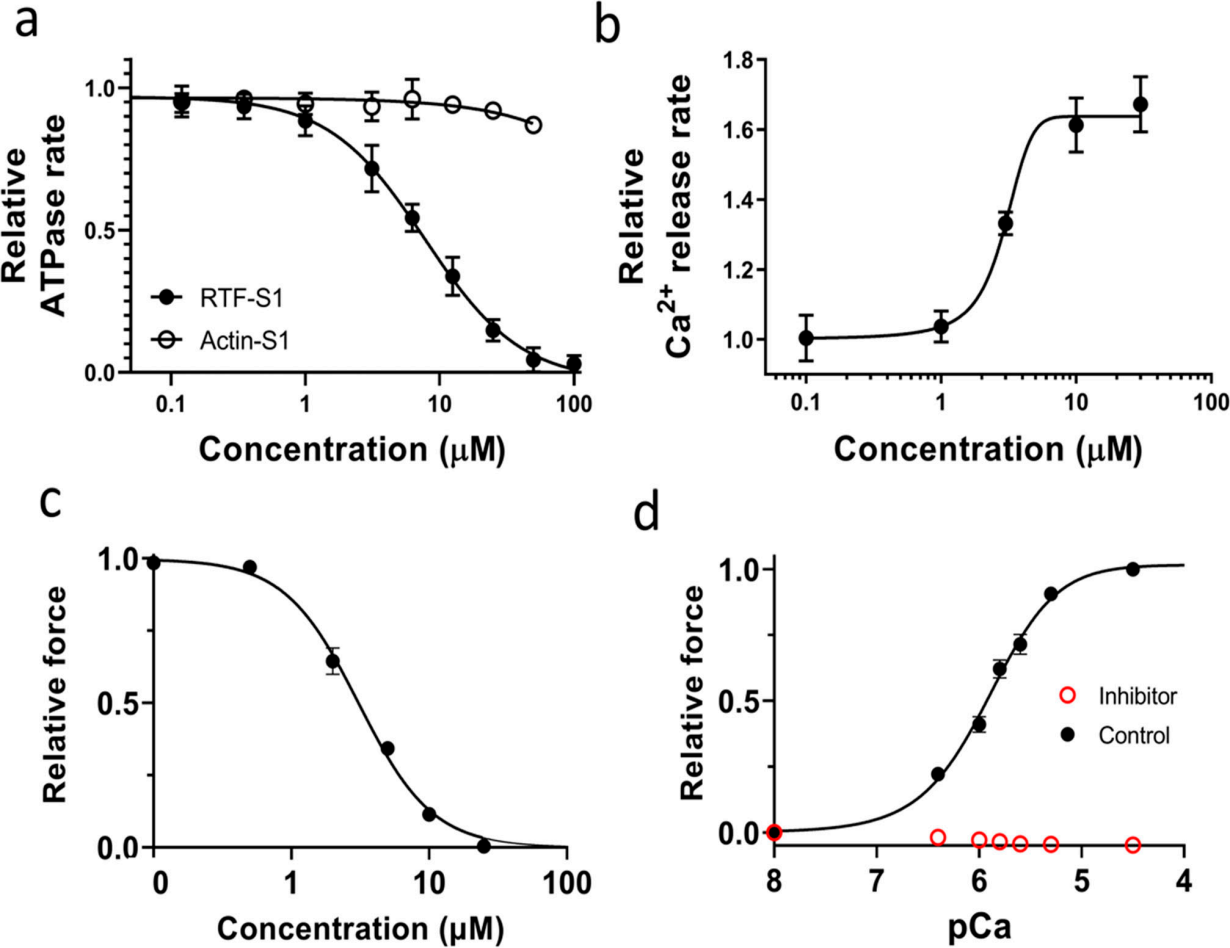
**Effects of the inhibitor (MYK-7660) on the chemo-mechanical activity of different sarcomere systems. (a)** The concentration-dependent steady-state ATPase activity (moles ATP used per second per mole of myosin S1 heads) of bovine cardiac myosin subfragment S1 with actin (open symbol) and with RTFs at pCa 6 (closed symbols). Inhibition (IC_50_ = 9 ± 3 μM) is specific to the RTF-S1 system, implicating that the compound inhibits the ATPase activity by shutting down the RTF system and not through actin and myosin. **(b)** Concentration-dependent transient kinetics of the Ca^2+^ release rate (s^−1^) from the RTF system. The AC_50_ of the increase in the Ca^2+^ release rate is measured as 3 ± 0.5 μM. **(c)** The concentration-dependent relative maximum force of permeabilized porcine myocardium. The IC_50_ of force inhibition is 3 ± 1 μM. IC_50_ and Hill slope parameters to the fits are given in Table S1. Lines in all plots are Hill fits to the data. **(d)** Relative force-pCa curve of permeabilized porcine myocardium in the absence or presence of 100 μM inhibitor (MYK-7660) during x-ray diffraction experiments. There is no detectable force increase above baseline generated by the myocardium in the presence of the inhibitor at all pCa values tested. Data are expressed as mean ± SEM (*n* = 2 for a and b; *n* = 6 for c; *n* = 12 in the control group; and *n* = 11 in the inhibitor group for d).

For ATPase measurements concerning the thin filament inhibitor (MYK-7660) in Fig. 1 a, actin-activated and regulated thin filament-activated ATPase measurements of the soluble S1-subfragment of the myosin molecule, as a function of increasing MYK-7660 concentrations (0–100 µM in the final buffer samples), are performed as described previously (Gollapudi et al., 2021).

Ca^2+^ dissociation rate from the RTF system in the presence of increasing MYK-7660 concentrations (0–100 µM in the final buffer samples) in Fig. 1 b is measured in a stopped-flow instrument (KinTek Model AutoSF-120) using transient kinetic measurements. Briefly, the RTFs (with a final concentration of 7 μM actin, 2 μM tropomyosin, and 2 μM troponin) are preincubated with ∼1 μM Ca^2+^ (pCa 6) and are rapidly mixed with a fluorescent Ca^2+^-chelator, Quin-2, of a final concentration of 50 μM in a buffer containing 20 mM Tris-HCL (pH 7.4), 10 mM KCl, 3 mM MgCl_2_, and 1 mM DTT. Experiments are performed at 25°C by exciting Quin-2 at 310 nm and monitoring the emission at 450 nm. Under these conditions, no significant contribution to the fluorescence is measured from MYK-7660 alone.

The dose-dependent maximally activated force data are collected in the presence of increasing MYK-7660 concentrations (0, 0.5, 2, 5, 10, and 25 μM in both pCa 8 and pCa 4.5 solutions) on skinned porcine myocardium (Fig. 1 c). The tissues are relaxed in pCa 8 solution with the same concentration of MYK-7660 as the following pCa 4.5 solution. Forces are normalized against the force generated with no inhibitor.

### Biochemical SRX measurements

Single ATP turnover kinetic experiments using a fluorescent 2′/3′-O-(*N*-Methylanthraniloyl) (mant)-ATP are conducted in a 96-well plate fluorescence plate reader at 25°C. A protocol involving this method has been described in previous studies (Gollapudi et al., 2021). This assay measures fluorescent nucleotide (excitation is at 385 nm, and emission is acquired using a long-pass filter with a cutoff at 450 nm) release rates following incubation of myosin preparations with mant-ATP and chased with excess unlabeled ATP. Briefly, in the first step, 100 μl of 0.8 μM myosin is combined with 50 μl of 3.2 μM mant-ATP in a UV-transparent fluorescence plate, and the reaction is aged for 60 s to allow binding and hydrolysis of mant-ATP to inorganic phosphate and mant-ADP. In the second step, mant-nucleotides are chased with non-fluorescent ATP by adding 50 μl of 16 mM non-fluorescent ATP to the above mixture, and the resulting fluorescence decay due to mant-nucleotide dissociation from myosin is monitored over time. The final buffer composition is as follows: 20 mM Tris-HCl (pH 7.4), 30 mM KCl, 1 mM EGTA, 3 mM MgCl_2_, and 1 mM DTT, unless otherwise mentioned. The ionic strength of this buffer is ∼39 mM, assuming negligible contribution from the buffer system. The concentrations of myosin, mant-ATP, and non-fluorescent ATP in the final mixture are 0.4 μM, 0.8 μM, and 4 mM, respectively. The fluorescence decay profile obtained during the chase phase characteristically depicts two phases, a fast phase followed by a slow phase. Therefore, a bi-exponential function is fitted to each trace to estimate four parameters corresponding to amplitudes and rates of the fast and slow phases. The fast and slow phases correspond to the myosin activity in the DRX and SRX states, respectively (Gollapudi et al., 2021).

### X-ray diffraction

X-ray diffraction experiments are performed at the BioCAT beamline 18ID at the Advanced Photon Source, Argonne National Laboratory (Fischetti et al., 2004). The x-ray beam energy is set to 12 keV (0.1033 nm wavelength) at an incident flux of ∼5 × 10^12^ photons per second. The specimen to detector distance is ∼3 m. The preparation is then attached to a hook on a force transducer (model 402B, Aurora Scientific, Inc.) and a static hook. The muscle is incubated in a customized chamber whose bottom is attached to a heat exchanger, so the solution is kept between 28 and 30°C. For remote solution changes, the chamber is connected to a multiway valve syringe pump (Hamilton model 500). The muscles are stretched to a sarcomere length of 2.3 µm using micromanipulators attached to the hooks while monitoring light diffraction patterns from a helium-neon laser (633 nm) on a screen. The x-ray patterns are collected sequentially at seven increasing Ca^2+^ concentrations (pCa 8, pCa 6.4, pCa 6, pCa 5.8, pCa 5.6, pCa 5.3, and pCa 4.5) in the absence or presence of 100 μM of MYK-7660, well below its solubility in the PBS buffer (180 μM), on a MarCCD 165 detector (Rayonix, Inc.) with a 1 s exposure time. To minimize radiation damage, the muscle samples are oscillated along their horizontal axes at a velocity of 1–2 mm/s. The irradiated areas are moved vertically after each exposure to avoid overlapping x-ray exposures. The force-pCa data are collected during the x-ray experiment (Fig. 1 d). Force measurements are normalized against the force generated at pCa 4.5 in the control experiment. There is no detectable increase in force above baseline in the inhibitor group in the presence of 100 μM MYK-7660.

### X-ray data analysis

**Figure S1.**
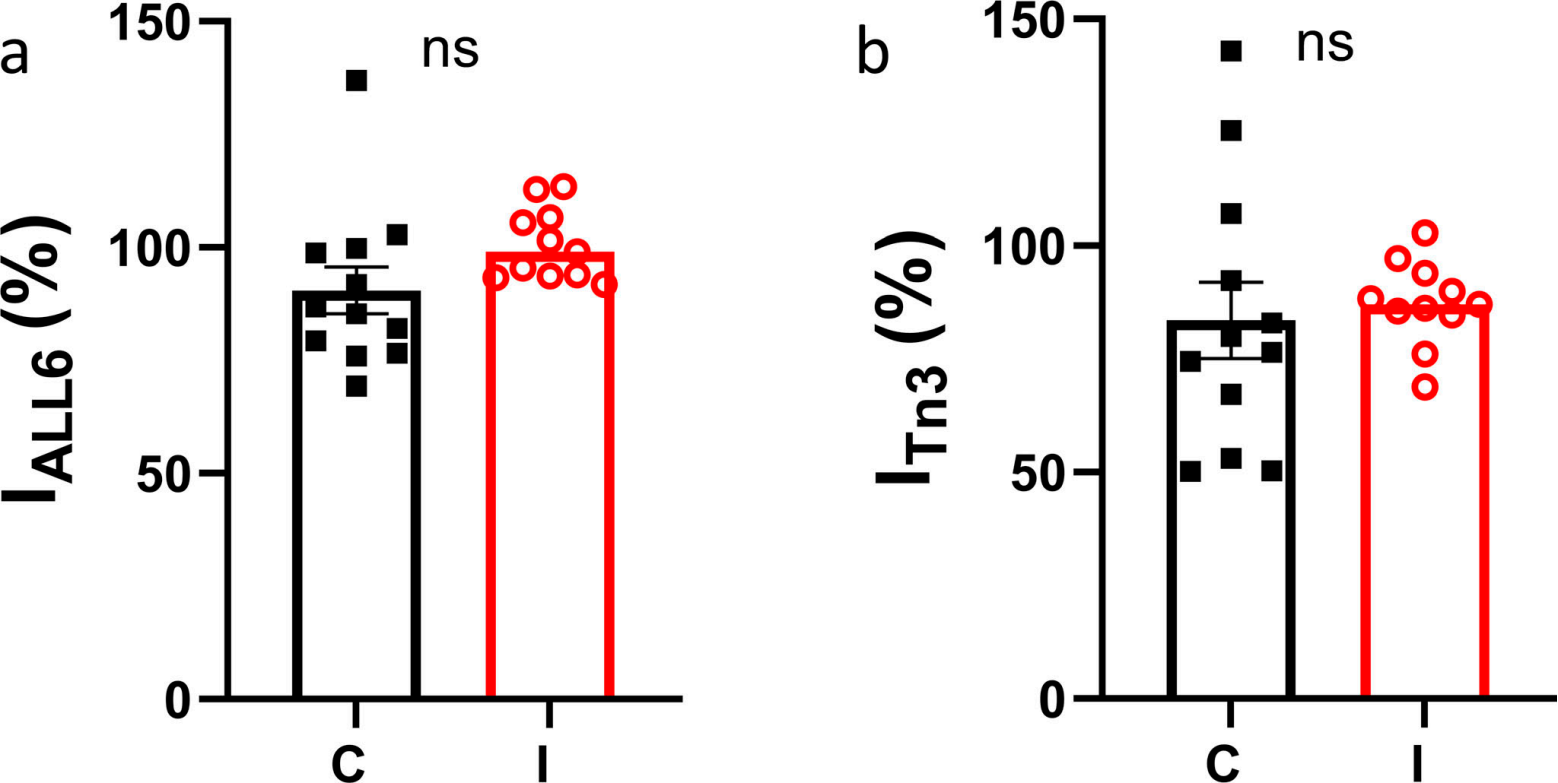
**Thin filament-based x-ray reflections in the presence and absence of inhibitor (MYK-7660).** **(a and b)** The intensity of the sixth-order actin-based layer line (a) and the third-order troponin meridional reflection (b) at pCa 4.5 normalized to its value at pCa 8 (C: control; I: inhibitor).

The data are analyzed using data reduction programs from the open-source MuscleX software package developed at BioCAT (Jiratrakanvong et al., 2018). One to two patterns are collected under each condition, and reflection spacings and intensities extracted from these patterns are averaged. As described previously, the equatorial reflections are measured by the “Equator” routine in MuscleX (Ma et al., 2018a). For subsequent analyses, the four quadrants, divided by meridian and equator, of x-ray patterns are averaged together to improve the signal-to-noise ratio, and the diffuse scatterings are subtracted from the x-ray diffraction patterns with the “Quadrant Folding” routine in MuscleX. The intensities and spacings of meridional and layer line reflections are measured by the “Projection Traces” routine in MuscleX, as described previously (Ma et al., 2020). The spacings of targeted reflections are estimated by measuring the distance from the beam center to the peak position as the centroid of the intensity in profile, considering only the top half of the diffraction peak (Huxley et al., 1994; Kiss et al., 2018). The center of mass of the cross-bridges (R_m_) is estimated based on modeling the layer line as a J3 Bessel function with the argument 4.2 = 2 × π × r × R_m_, where r is a reciprocal space coordinate as described (Ma et al., 2021; Ma and Irving, 2022). To compare the intensities under different conditions, the measured intensities of x-ray reflections are normalized to the sixth-order actin-based layer line intensities. This reflection is chosen because its intensity did not change significantly from its value at pCa 8 with changes of contractile state in cardiac muscle (see Fig. S1)

### Statistics

**Figure 2.**
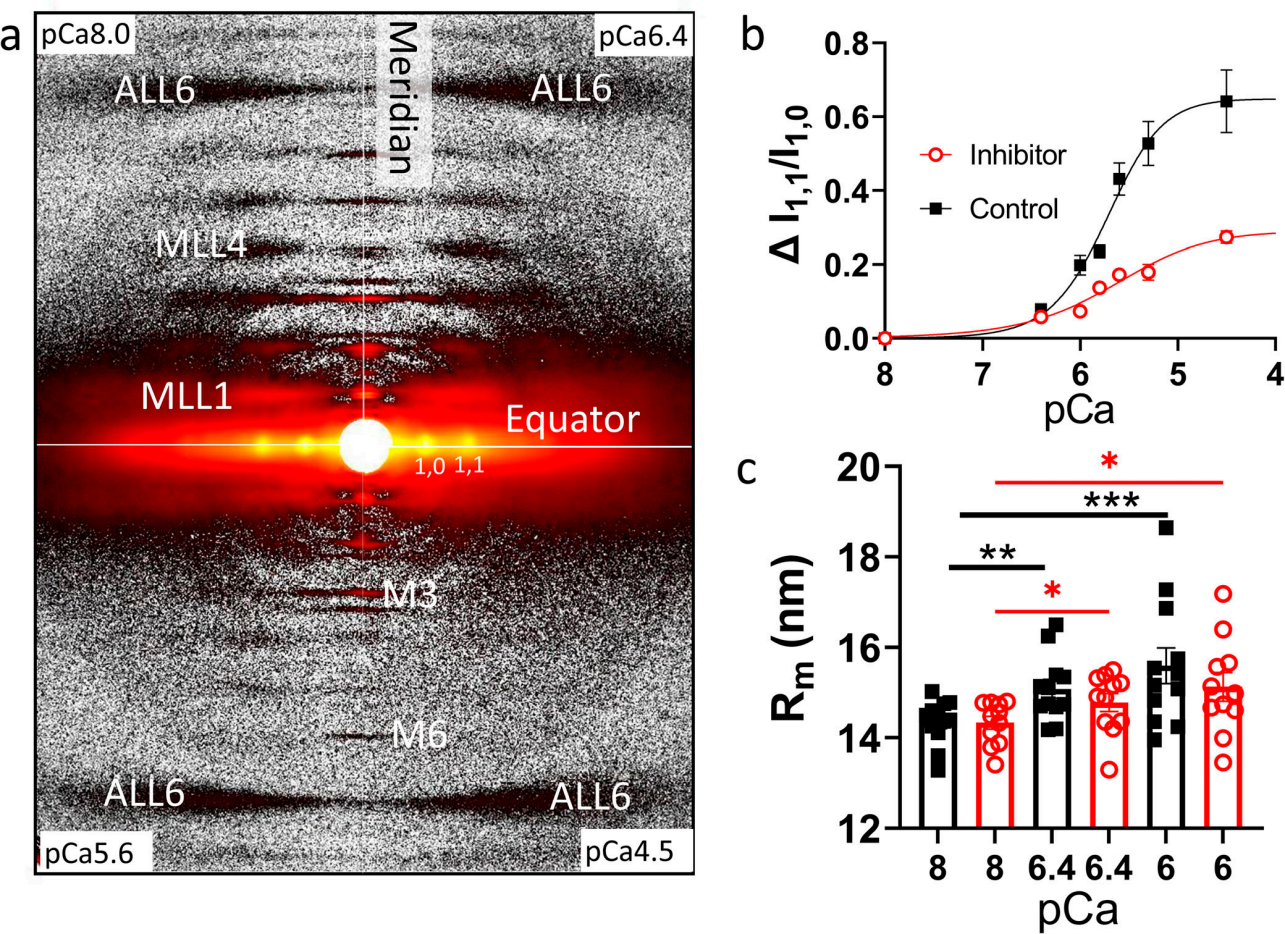
**Myosin heads move radially away from the thick filament backbone. (a)** Representative x-ray diffraction patterns from permeabilized porcine myocardium in different Ca^2+^ in the absence of force. **(b)** Change of equatorial intensity ratio (Δ I_1,1_/I_1,0_) at different Ca^2+^concentrations in the presence (red) and absence (black) of inhibitor (MYK-7660). **(c)** The radius of the average mass of myosin heads (R_m_) at different Ca^2+^ concentrations in the presence (red) and absence (black) of inhibitor. Ca^2+^ shifts the distribution of myosin heads away from the thick filament backbone towards the thin filaments. Data are expressed as mean ± SEM (*n* = 12 in the control group and *n* = 11 in the inhibitor group).

**Figure 3.**
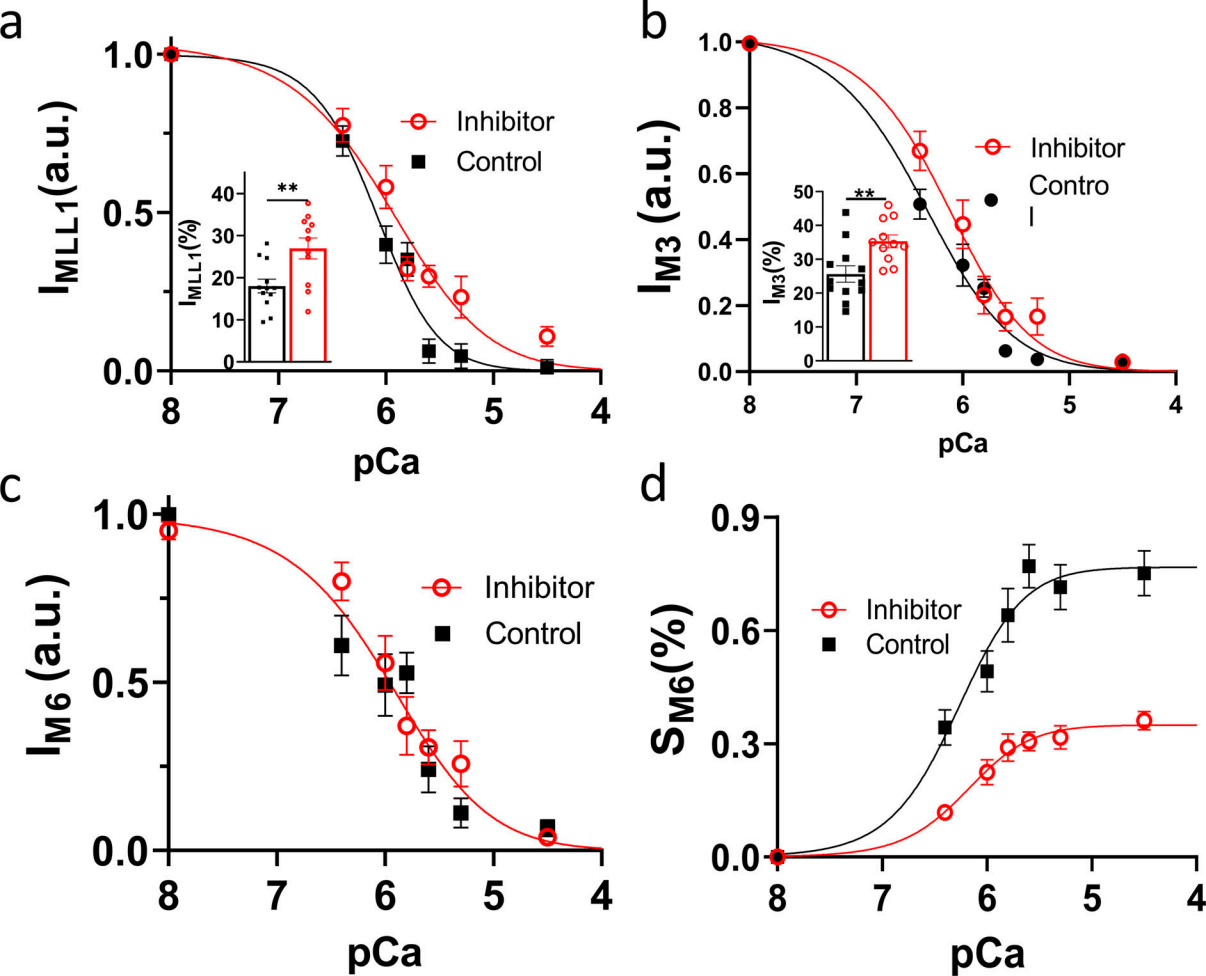
**Thick filament structure changes in the presence of Ca**^**2+**^**.** **(a and b)** The intensity of first-order myosin-based layerline (a) and third-order of myosin-based meridional reflection (b) in different concentrations of Ca^2+^ in the presence (red) and absence (black) of inhibitor (MYK-7660). **(c and d)** The intensity (c) and spacing (d) of the sixth-order of myosin-based meridional reflection in different Ca^2+^ concentrations in the presence (red) and absence (black) of inhibitor. Ca^2+^ reduces the proportion of myosin heads in ordered states on the thick filament and induces structural changes in the thick filament backbone. Data are expressed as mean ± SEM (*n* = 12 in the control group and *n* = 11 in the inhibitor group).

**Figure 4.**
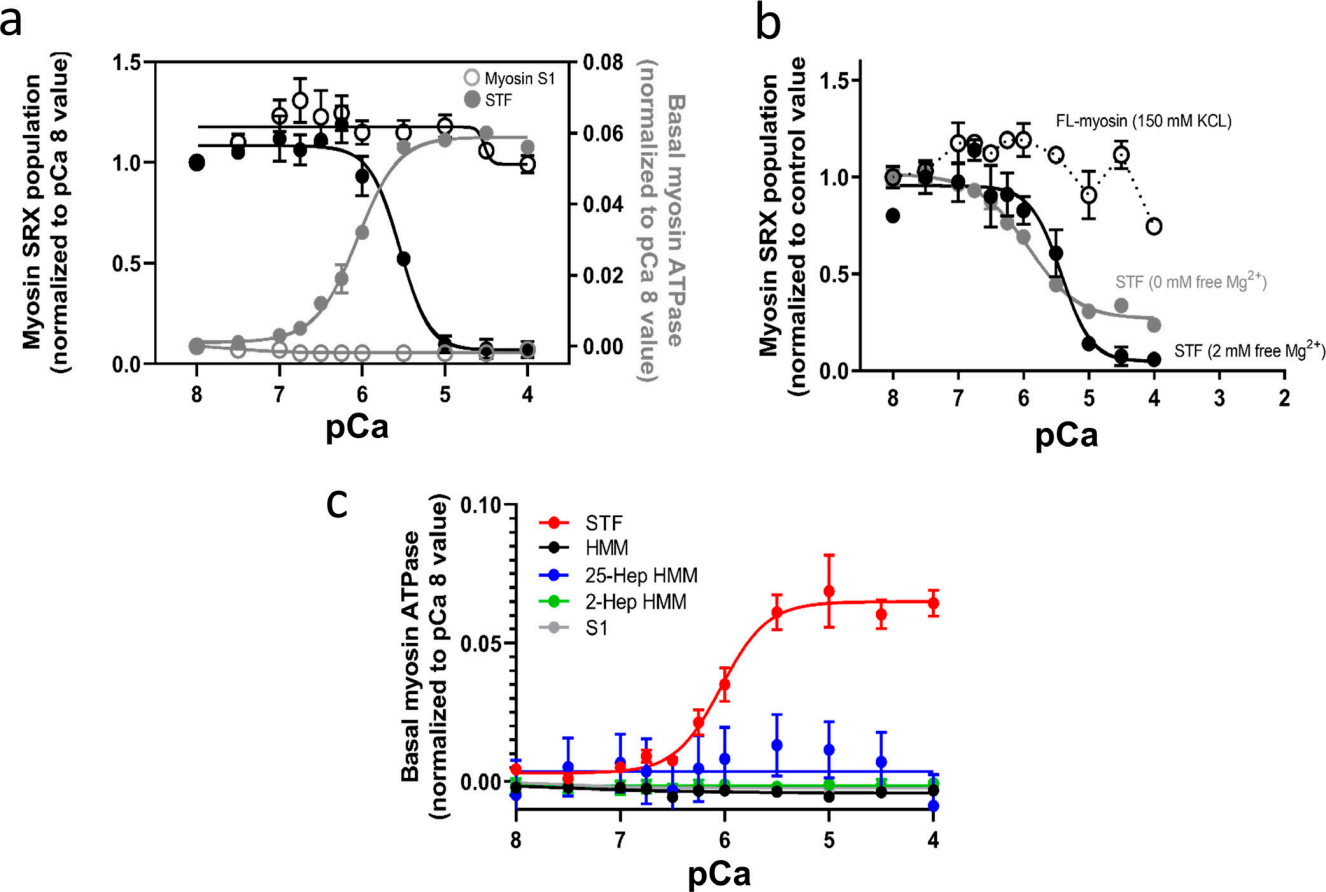
**The biochemical SRX states of myosin in reconstituted STF are modulated by Ca**^**2+**^**. (a)** Normalized myosin SRX population (expressed as a fraction of the initial %SRX at pCa 8; black symbols) and normalized basal myosin ATPase activity (expressed as [ATPase value − ATPase value at pCa 8]; grey symbols) in STF reconstituted from bovine cardiac full-length myosin (solid symbols) and bovine cardiac myosin S1-subfragment (open symbols) at different Ca^2+^ concentrations. **(b)** Normalized myosin SRX population in STF formed from full-length myosin at 30 mM KCl (0 mM free Mg^2+^, dark grey; and 2 mM free Mg^2+^, black) and 150 mM KCl (open circles and solid symbols) at different Ca^2+^ concentrations. Ca^2+^ destabilizes the biochemical myosin SRX state(s) only when assembled into thick filaments. **(c)** Normalized basal myosin ATPase activity (expressed as [ATPase value − ATPase value at pCa 8]; in STF reconstituted from bovine cardiac full-length myosin (red), bovine cardiac HMM (black), and subfragment-S1 (grey), and in recombinant human cardiac 2-hep (green) and 25-hep HMM (blue) at different Ca^2+^ concentrations. All data are expressed as mean ± SEM (*n* ≥ 9 from three independent experiments that used at least two different preparations of the same reagent purification). Raw data at pCa 8 and pCa 4 are provided in Table 1.

Statistical analyses are performed using GraphPad Prism 9 (GraphPad Software). The results are given as mean ± SEM. One-way repeated measures ANOVA with the Geisser-Greenhouse correction and Tukey’s multiple comparisons test with individual variances computed for each comparison is performed on bar graphs in Fig. 2 c. A nested *t-*test was performed for the bar graphs in Fig. 3 and Fig. S1. The relative changes versus pCa curves are fit to a four-parameter modified Hill equation (minimum response + [maximum response − minimum response] / [1 + 10^h^ (pCa_50_ − pCa)]; Walker et al., 2010), where pCa_50_ is the Ca^2+^ concentration yielding a response halfway between the minimum and maximum values reported in the article. Symbols on figures: *: P < 0.05, **: P < 0.01, ***: P < 0.001. For Fig. 4, each experiment is repeated at least twice with a minimum of two replicates per experiment, and a two-tailed Student’s *t*-test is used to differentiate the changes in parameters among groups (P < 0.01).

### Online supplemental material

Fig. S1 shows thin filament-based x-ray reflections in the presence and absence of the inhibitor (MYK-7660). Table S1 includes parameters obtained from MYK-7660 characterization. Table S2 includes parameters obtained from fitting x-ray datasets to the modified Hill equation. Supplemental text at the end of the PDF provides additional information about Ca^2+^-induced thin filament reflections in the presence and absence of MYK-7660.

## Results

### Characterization of MYK-7660

Here, we use a small-molecule inhibitor of the thin filament system (MYK-7660) to decouple Ca^2+^-mediated thin filament–based regulation from thick filament–based regulation. Actin-activated (Actin-S1) and regulated thin filament (RTF-S1)–activated ATPase activity at pCa 6 of bovine cardiac myosin subfragment S1 was measured in response to increasing concentrations of the inhibitor (MYK-7660; Fig. 1 a and Table S1). The ATPase rates were normalized against the basal ones in the absence of the drug for these two systems, 0.02 ± 0.002 s^−1^ (*n* = 3). The compound inhibited the RTF-S1 system (IC_50_ = 9 ± 3 μM) but not the Actin-S1 in a dose-dependent manner, suggesting that the mechanism of inhibition is through shutting down the regulated thin filament system and not through actin and myosin. The Ca^2+^ release rate is enhanced as the concentration of MYK-7660 increases (AC_50_ = 3 ± 0.5 μM; Fig. 1 b and Table S1). This can explain the ATPase inhibition of MYK-7660 without hampering Ca^2+^ binding to troponin.

The inhibitor inhibited the force production of permeabilized porcine myocardium in a dose-dependent manner with an IC_50_ of 3 ± 1 (Fig. 1 c and Table S1; *n* = 6). The force dropped to zero beyond 25 μM inhibitor concentration. The force produced by permeabilized porcine myocardium at different pCa values during the x-ray experiment is presented in Fig. 1 d. Permeabilized porcine myocardium produces a classic sigmoidal force-pCa curve at a sarcomere length of 2.3 μm in the absence of the inhibitor with a pCa_50_ of 5.91 ± 0.1 (Fig. 1 d, black symbols; Table S2; *n* = 12). However, in the presence of saturating levels of the inhibitor (100 μM), no active contraction is detected at all pCa values (Fig. 1 d, red symbols; *n* = 11). 100 μM of MYK-7660 was chosen for all x-ray diffraction experiments to ensure complete inhibition of active force.

### Radial movement of myosin heads

Small-angle x-ray diffraction is used to examine the structural transitions of permeabilized porcine cardiac myocardium at different Ca^2+^ concentrations in the presence of the inhibitor. At pCa 8 the x-ray diffraction patterns of permeabilized porcine cardiac myocardium show characteristic relaxed patterns similar to those reported previously (Anderson et al., 2018; Ma et al., 2021). With increasing Ca^2+^ concentration, the intensities of all the myosin-based reflections (M3, M6, MLLs in Fig. 2 a) become weaker. The intensities of the sixth-order actin-based layer line (ALL6, Fig. 2 a) are relatively stable throughout the experiment indicating that the changes in the thick filament myosin-based reflections are due to the direct effects of Ca^2+^-binding and not a result of radiation damage or by strong binding of cross-bridges to the thin filament.

The equatorial intensity ratio (I_1,1_/I_1,0_) is indicative of the proximity of myosin heads to actin in relaxed muscle and is closely correlated to the number of force-producing cross-bridges in activated muscle (Haselgrove and Huxley, 1973; Matsubara, 1980; Ma et al., 2018a; Ma et al., 2020). The change of I_1,1_/I_1,0_ (Δ I_1,1_/I_1,0_) versus pCa shows a sigmoidal shape with a pCa_50_ of 5.7 (5.5–5.9 for 95% CI) for the control group (*n* = 12). Surprisingly in the inhibitor group (*n* = 11), where active contractions are eliminated, I_1,1_/I_1,0_ also progressively increases with increasing Ca^2+^ concentration (Fig. 2 b and Table S2), with a pCa_50_ of 5.6 (3.5–5.9 for 95% CI), although the amplitudes of the change are smaller compared to the control group. The radius of the center of mass of the cross-bridges (R_m_), which directly measures the proximity of helically ordered myosin heads to the thick filament backbone (Ait-Mou et al., 2016; Ma et al., 2018a), increases from 14.34 ± 0.14 nm at pCa 8–15.59 ± 0.4 nm at pCa 6 in the control group, R_m_ increases similarly in the presence of the inhibitor (14.34 ± 0.14 at pCa 8, to 15.13 ± 0.32 nm at pCa 6; Fig. 2 c). The ΔI_1,1_/I_1,0_ and R_m_ data indicate that with increasing Ca^2+^ concentration, myosin heads move radially away from the thick filament backbone under conditions where they cannot bind to thin filaments, suggesting a regulatory role of Ca^2+^ directly on the thick filament.

### Structural *off*-to-*on* transitions

In a resting muscle, most myosin heads are quasi-helically ordered on the surface of the thick filament, where these *off*-state myosin heads produce the myosin-based layer line reflections. Myosin heads lose their helical order when turned *on* to participate in contraction (Huxley and Brown, 1967; Ma and Irving, 2022). The intensity of the first-order myosin-based layer line (I_MLL1_) and the third-order myosin-based meridional reflection (I_M3_), both of which correlate with the ordering of myosin heads (Reconditi, 2006; Ma and Irving, 2022), decreases progressively in the presence of increasing Ca^2+^. The I_MLL1_ reflection changes (Fig. 3 a and Table S2) with a pCa_50_ of 6.05 (5.91–6.19 for 95% CI) and 6.09 (5.98–6.19 for 95% CI) for the inhibitor and control group, respectively, whereas the I_M3_ reflection changes (Fig. 3 b and Table S2) with a pCa_50_ of 6.17 (6.03–6.33 for 95% CI) and 6.36 (6.24–6.51 for 95% CI) for these two experimental groups, respectively. Compared to pCa 8, the I_MLL1_ and I_M3_ intensities at pCa 4.5 decrease to 27 ± 2.5% and 35 ± 1.9%, respectively, in the inhibitor group, whereas it decreases to 18 ± 1.6% and 26 ± 2.4% in the control group (inset in Fig. 3, a and b).

I_MLL1_ and I_M3_ data show that myosin heads lose their helical ordering in the presence of Ca^2+^, which strongly indicates switching from the ordered *off* states to the disordered *on* states in cardiac muscle thick filaments. The diminished I_MLL1_ and I_M3_ intensities at pCa 4.5 and the leftward shift of the intensity decay curve in the control group indicate that active tension can activate the residual myosin heads on the thick filament backbone as expected from the mechano-sensing thick filament activation mechanism. However, the majority of the loss of ordering in the myosin heads can be accounted for by Ca^2+^-mediated activation of the thick filament in the absence of cross-bridge forces.

The decay of the intensity of the sixth-order myosin-based meridional reflection (I_M6_), which arises primarily from the thick filament backbone (Reconditi, 2006), with increasing Ca^2+^ concentration is indistinguishable between the inhibitor and control groups (P = 0.6) with a similar pCa_50_ of 5.96 (5.78–6.13 for 95% CI; Fig. 3 c and Table S2). Currently, there are no obvious mechanistic explanations for the decay in I_M6_ in the presence of Ca^2+^ and the absence of force. Since I_M6_ arises primarily from the thick filament backbone, the decrease of I_M6_ in the presence of Ca^2+^ suggests a structural change in the thick filament backbone induced by Ca^2+^, independent of thick filament strain. The spacing of the M6 reflection (S_M6_), which reports the periodicity of the thick filament backbone (Ma and Irving, 2022), increases to 0.73 ± 0.06% at the fully activated state (pCa 4.5) in the control group and 0.36 ± 0.02% in the inhibitor group where active force is absent (Fig. 3 d and Table S2). While the larger S_M6_ changes in the control group are caused by both the release of *off* states myosin heads and the increase in strain on the thick filament by active contraction (Reconditi et al., 2019), the increase in S_M6_ in the absence of active force suggests that an, at least partial, *off*-to-*on* transition of the thick filament can occur solely in response to Ca^2+^.

### SRX to DRX transitions

Given our observation of Ca^2+^-induced *off*-to-*on* transitions of the thick filament in the absence of active force, and inspired by previous work suggesting that there might be direct effects of Ca^2+^ on thick filaments (Morimoto and Harrington, 1974; Metzger and Moss, 1992; Podlubnaya et al., 2000), we explored whether these structural transitions can be translated into functional alterations. The thin filament inhibitor (MYK-7660) we used in this study has fluorescence in the 400–500 nm range, making performing conventional SRX/DRX assays on muscle tissue impractical. To test our hypothesis, since our results suggest that myosin or myosin filaments are the direct targets of Ca^2+^ binding, we turned our focus to reconstituted cardiac STF, which can faithfully capture SRX/DRX transitions under various conditions (Gollapudi et al., 2021).

The basal myosin SRX population in the STF system at pCa 8 is 15 ± 5%, which progressively decreases (P < 0.01) to 3 ± 2% (pCa_50_ of 5.5; 5.4–5.7 for 95% CI) at pCa 4 (Fig. 4 a [normalized data], solid black symbol) with no considerable change in the ATPase cycling rate of the SRX states (*n* = 5). This destabilization of the SRX states, which presumably populates myosin in some DRX states, along with an increase in the cycling rates of the DRX population, leads to an overall Ca^2+^-dependent increase (P < 0.01) in the steady-state basal myosin ATPase activity from 0.03 ± 0.01 s^−1^ at pCa 8 to 0.09 ± 0.02 s^−1^ at pCa 4 with a pCa_50_ of 6.1 (5.9–6.2 for 95% CI; Fig. 4 a [normalized data], solid grey symbol; *n* = 3). Further investigation with the soluble S1-subfragment of the myosin molecule (Fig. 4 a [normalized data], open symbols, unnormalized data shown in Table 1) and full-length myosin at high ionic strengths (150 mM KCl; Fig. 4 b) that do not fully form filamentous thick filaments demonstrates a loss of this Ca^2+^-dependent modulation, suggesting that the Ca^2+-^binding mechanism may not be intrinsic to myosin S1 or isolated full-length myosin but rather involve only myosins forming filamentous structures. This result is further supported by a lack of Ca^2+^-dependent activation of the ATPase in different soluble double-headed HMM constructs containing variable lengths of the S2-subfragment (Fig. 4 c and Table 1) but lacks the light meromyosin (LMM) domain that can assemble myosin into bipolar thick filaments. For example, the steady-state basal myosin ATPase activity in the STF system at pCa 8 is 0.03 ± 0.01 s^−1^, which increases to 0.1 ± 0.02 s^−1^ at pCa 4 with a pCa_50_ of 6.0 (5.9–6.1 for 95% CI; Fig. 4 c [normalized data], red symbols; Table 1). However, such Ca^2+^-mediated ATPase activation is absent in enzymatically produced bovine cardiac HMM (black symbols) and S1 (grey symbols) and recombinantly produced human cardiac 2-hep (green symbols) and 25-hep HMM (blue symbols), suggesting that the Ca^2+^-mediated regulation mechanism may not be intrinsic to soluble myosin constructs but rather involve only myosins forming filamentous structures.

**Table 1.** Parameters obtained from biochemical assays

	Raw values at pCa 8	Raw values at pCa 4	pCa_50_ (95% CI)	Hill slope (95% CI)
STF SRX (Fig. 4 a)	15 ± 5%	3 ± 2%	5.5 (5.4–5.7)	2.4 (1.4–2.5)
S1 SRX (Fig. 4 a)	13 ± 2%	12 ± 3%	N/A	N/A
STF ATPase (Fig. 4 a)	0.03 ± 0.01 s^−1^	0.09 ± 0.02 s^−1^	6.1 (6.0–6.1)	1.7 (1.3–2.3)
S1 ATPase (Fig. 4 a)	0.03 ± 0.01 s^−1^	0.03 ± 0.01 s^−1^	N/A	N/A
STF SRX (2 mM Mg^2+^; Fig. 4 b)	17 ± 2%	1 ± 2%	5.4 (5.2–5.7)	1.8 (0.9–1.9)
STF SRX (0 mM Mg^2+^; Fig. 4 b)	13 ± 1%	3 ± 2%	5.9 (5.8–6.1)	1.1 (0.8–1.5)
FL-myosin SRX (150 mM KCl; Fig. 4 b)	5 ± 3%	4 ± 2%	N/A	N/A
STF ATPase (Fig. 4 c)	0.03 ± 0.01 s^−1^	0.1 ± 0.02 s^−1^	6.0 (5.9–6.1)	1.9 (1.4–3.1)
HMM ATPase (Fig. 4 c)	0.03 ± 0.05 s^−1^	0.03 ± 0.05 s^−1^	N/A	N/A
25-hep HMM ATPase (Fig. 4 c)	0.1 ± 0.05 s^−1^	0.1 ± 0.05 s^−1^	N/A	N/A
2-hep HMM ATPase (Fig. 4 c)	0.08 ± 0.05 s^−1^	0.07 ± 0.05 s^−1^	N/A	N/A
S1 ATPase (Fig. 4 c)	0.03 ± 0.05 s^−1^	0.03 ± 0.05 s^−1^	N/A	N/A

An EF-hand motif in the myosin's RLC domain has been shown to bind magnesium (Mg^2+^) under relaxed conditions and is increasingly occupied by Ca^2+^ as its concentration increases during muscle contraction (Markandran et al., 2021). We next studied the Ca^2+^-dependent effect in the absence and presence of saturating amounts of free Mg^2+^ bound to the RLC to find that the Ca^2+^-dependent destabilization of the myosin SRX is unaltered under these two conditions (Fig. 4 b), suggesting that the RLC EF-hand Ca^2+^-binding motif is not the primary Ca^2+^ transducer responsible for *off-*to-*on* transitions in the thick filament. The primary effect of Ca^2+^ on the RLC regarding thick filament *off*-to-*on* transitions appears to be indirect through MLCK phosphorylation (Stull et al., 2011), which is not present in our experimental systems. Binding of Ca^2+^ to a different site in the RLC or elsewhere on myosin cannot be excluded based on our data. Altogether, these data suggest that this Ca^2+^-dependent modulation of myosin is a feature that is unique to myosins assembled into thick filaments.

## Discussion

In the x-ray study of permeabilized tissue, although the thin filament is shut down by the small-molecule inhibitor (MYK-7660), other sarcomeric proteins such as myosin-binding protein C and titin are present and known to bind to Ca^2+^ (Labeit et al., 2003). However, the biochemical studies with cardiac synthetic thick filament reconstituted from purified full-length myosin showing that Ca^2+^ can bind and destabilize the SRX states of myosin, in the absence of these accessory proteins, strongly indicates that a Ca^2+^-dependent switch is an intrinsic property of myosin. At this time, the Ca^2+^-binding site(s) on the thick filament has not been identified, nor can we exclude other, less specific mechanisms. Since Ca^2+^ only turns myosin on when they are in filaments, and we observe structural transitions in the thick filament backbone upon Ca^2+^ binding, we strongly suspect that the Ca^2+^ binding to myosin as packed in bipolar filaments may relieve a head–backbone interaction that holds myosin heads in *off* states at diastolic Ca^2+^ concentrations. These head–backbone interactions are widely assumed to involve heads in the “interacting heads motif,” but this may not necessarily be the case in all circumstances (Craig and Padron, 2022).

**Figure 5.**
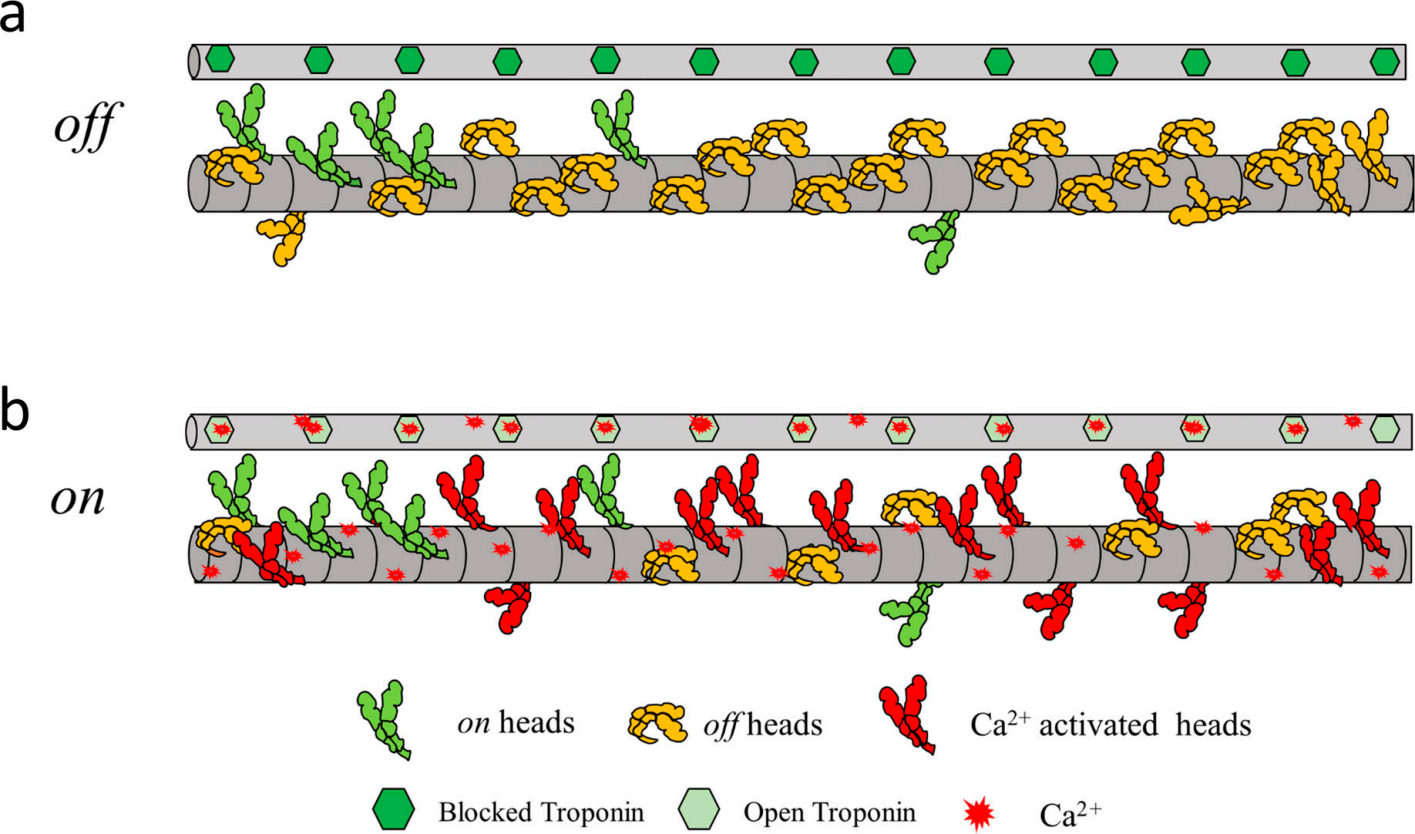
**Ca**^**2+**^**-mediated dual filament regulation mechanism in cardiac muscle.** A schematic cartoon of the cardiac thin (thin grey bar) and thick filament (thick grey bar; not drawn to scale) showing the organization of the regulated thin filament and myosin. **(a)** In the absence of Ca^2+^, the thin and thick filaments in resting muscle are in the deactivated (*off*) states, and individual myosin heads may be in either the *off* (orange) or the *on* (green) state(s). **(b)** Ca^2+^ (red) can independently bind to the thick and thin filament and activate them simultaneously. Ca^2+^-mediated regulation of the thick filament destabilizes the myosin heads from the *off* states (orange) to the *on* states (red heads), along with the already *on* heads (green) which allows the swinging of the S2-subfragment that facilitates myosin heads to bind to actin.

The results presented above lead to a novel concept of a Ca^2+^-mediated dual-filament regulation model in cardiac muscle (Fig. 5). At low Ca^2+^ concentration during diastole, the thick and thin filaments are in *off* states. Ca^2+^ from extracellular influx upon arrival of an action potential, released from sarcoplasmic reticulum through a Ca^2+^-induced Ca^2+^-release mechanism (Eisner et al., 2017), binds to both thin and thick filaments and allows the heart muscle to contract. The Ca^2+^ concentration can regulate the level of activation of both thin and thick filaments for them to work synergistically. The reuptake of Ca^2+^ after contraction will deactivate both thick and thin filaments. For example, a recent modeling study hypothesized a biochemically defined Ca^2+^-dependent “parked” state(s), where myosin heads are unable to bind actin, analogous to our structurally defined *off* state. This model could explain both activation and relaxation in twitches in the myocardium, providing more realistic relaxation rates, resting tensions, and myosin cross-bridge detachment rate than in other current models (Mijailovich et al., 2021). Notably, the Ca^2+^-mediated thick filament structural transitions shown in Figs. 2 and 3 and the functional transitions shown in Fig. 4 have a pCa_50_ in the range of 5.5 to 5.9, similar to the pCa_50_ of the force pCa curve (5.91 ± 0.1, Fig. 1 d), close the physiological range of systolic Ca^2+^ concentrations (0.3–3 µM [Sankaranarayanan et al., 2017]).

Additionally, there is a growing understanding that increased mitochondrial Ca^2+^ can augment ATP production (Finkel et al., 2015). These results indicate that evolution might have found an effective way to modulate cardiac muscle activation and relaxation by synchronizing both the thin and thick filament of the sarcomere and the energy supply by a single messenger, Ca^2+^. Imbalance in any of these components (Ca^2+^ flux, thin- or thick-filament Ca^2+^ sensitivity, and the roles of titin and MyBP-C) could disrupt the exquisite equilibrium of the system, leading to compensatory effects that cause long-term damage. We have, at this time, many unanswered questions. For example, is Ca^2+^-mediated thick filament activation, demonstrated here in steady-state measurements, fast enough to happen on a beat-to-beat basis? Is this activation sensitive to sarcomere length and relevant to length-dependent activation? These questions provide motivation for future experiments.

Ca^2+^ plays a central role in cardiac muscle function, but its role has been traditionally attributed to thin filament-based regulation inside the sarcomere. Our discovery of a direct Ca^2+^-mediated destabilization of the *off* states myosin on cardiac thick filaments warrants a reconstruction of previous understandings of the roles of Ca^2+^, including Ca^2+^ sensitivity and Ca^2+^ handling, in cardiac muscle in health and disease. Dysregulation of thick filament-based activation mechanisms appears to be the basis of many cardiomyopathies, including hypertrophic cardiomyopathy, which is hypothesized to be due to increased release of myosin heads from the sequestered *off* to *on* states and thereby increasing force-producing cross-bridges (Spudich, 2019; Nag and Trivedi, 2021), leading to hypercontractility and diastolic impairment. This understanding has led to a search for myosin inhibitors that mitigate *off* and *on* states dysregulation. The best known of these is mavacamten (Green et al., 2016), which has been shown by x-ray diffraction (Anderson et al., 2018; Ma et al., 2021) to enrich the population of *off-*state myosin heads. Therefore, the newly discovered Ca^2+^-mediated thick-filament regulation of force generation in cardiac muscle may need to be considered in developing future sarcomere-based drug modalities. Additionally, this Ca^2+^-mediated regulation of thick filaments, observed here in cardiac muscle, may turn out to be a fundamental component of all human skeletal systems, which opens the possibility of new therapeutic approaches for many congenital myopathies caused by sarcomeric protein mutations. More generally, insofar a significant fraction of the ATPase activity of the skeletal muscle myosin is to maintain temperature homeostasis in homeothermic organisms (Cooke, 2011), this new role of calcium as a driver of myosin ATP consumption may provide a unique perspective on the energetic costs of thermo-regulation.

## Supplementary Material

Table S1 include parameters obtained from MYK-7660 characterization

Table S2 include parameters obtained from fitting X-ray datasets to modified Hill equation

## Data Availability

The datasets generated or analyzed during this study are included in this article. The raw data are available from the corresponding authors upon reasonable request. The thin filament inhibitor MYK-7660 can be made available from Bristol Myers Squibb under a material transfer agreement or in collaboration with their scientists.

## Data availability

The datasets generated or analyzed during this study are included in this article. The raw data are available from the corresponding authors upon reasonable request. The thin filament inhibitor MYK-7660 can be made available from Bristol Myers Squibb under a material transfer agreement or in collaboration with their scientists.

## References

[bib1] Ait-Mou, Y., K. Hsu, G.P. Farman, M. Kumar, M.L. Greaser, T.C. Irving, and P.P. de Tombe. 2016. Titin strain contributes to the Frank-Starling law of the heart by structural rearrangements of both thin- and thick-filament proteins. Proc. Natl. Acad. Sci. USA. 113:2306–2311. 10.1073/pnas.1516732113 26858417 PMC4776536

[bib2] Alsulami, K., and S. Marston. 2020. Small molecules acting on myofilaments as treatments for heart and skeletal muscle diseases. Int. J. Mol. Sci. 21:9599. 10.3390/ijms21249599 PMC7767104 33339418

[bib3] Anderson, R.L., D.V. Trivedi, S.S. Sarkar, M. Henze, W. Ma, H. Gong, C.S. Rogers, J.M. Gorham, F.L. Wong, M.M. Morck, . 2018. Deciphering the super relaxed state of human beta-cardiac myosin and the mode of action of mavacamten from myosin molecules to muscle fibers. Proc. Natl. Acad. Sci. USA. 115:E8143–E8152. 10.1073/pnas.1809540115 30104387 PMC6126717

[bib4] Bagur, R., and G. Hajnoczky. 2017. Intracellular Ca(2+) sensing: Its role in calcium homeostasis and signaling. Mol. Cell. 66:780–788. 10.1016/j.molcel.2017.05.028 28622523 PMC5657234

[bib5] Caremani, M., F. Pinzauti, J.D. Powers, S. Governali, T. Narayanan, G.J.M. Stienen, M. Reconditi, M. Linari, V. Lombardi, and G. Piazzesi. 2019. Inotropic interventions do not change the resting state of myosin motors during cardiac diastole. J. Gen. Physiol. 151:53–65. 10.1085/jgp.201812196 30510036 PMC6314382

[bib6] Cooke, R. 2011. The role of the myosin ATPase activity in adaptive thermogenesis by skeletal muscle. Biophys. Rev. 3:33–45. 10.1007/s12551-011-0044-9 21516138 PMC3064898

[bib7] Craig, R., and R. Padron. 2022. Structural basis of the super- and hyper-relaxed states of myosin II. J. Gen. Physiol. 154:e202113012. 10.1085/jgp.202113012 34889960 PMC8669498

[bib8] Eisner, D.A., J.L. Caldwell, K. Kistamas, and A.W. Trafford. 2017. Calcium and excitation-contraction coupling in the heart. Circ. Res. 121:181–195. 10.1161/CIRCRESAHA.117.310230 28684623 PMC5497788

[bib9] Finkel, T., S. Menazza, K.M. Holmstrom, R.J. Parks, J. Liu, J. Sun, J. Liu, X. Pan, and E. Murphy. 2015. The ins and outs of mitochondrial calcium. Circ. Res. 116:1810–1819. 10.1161/CIRCRESAHA.116.305484 25999421 PMC6296495

[bib10] Fischetti, R., S. Stepanov, G. Rosenbaum, R. Barrea, E. Black, D. Gore, R. Heurich, E. Kondrashkina, A.J. Kropf, S. Wang, . 2004. The BioCAT undulator beamline 18ID: A facility for biological non-crystalline diffraction and X-ray absorption spectroscopy at the advanced photon source. J. Synchrotron Radiat. 11:399–405. 10.1107/S0909049504016760 15310956

[bib11] Gollapudi, S.K., M. Yu, Q.F. Gan, and S. Nag. 2021. Synthetic thick filaments: A new avenue for better understanding the myosin super-relaxed state in healthy, diseased, and mavacamten-treated cardiac systems. J. Biol. Chem. 296:100114. 10.1074/jbc.RA120.016506 33234590 PMC7948491

[bib12] Green, E.M., H. Wakimoto, R.L. Anderson, M.J. Evanchik, J.M. Gorham, B.C. Harrison, M. Henze, R. Kawas, J.D. Oslob, H.M. Rodriguez, . 2016. A small-molecule inhibitor of sarcomere contractility suppresses hypertrophic cardiomyopathy in mice. Science. 351:617–621. 10.1126/science.aad3456 26912705 PMC4784435

[bib13] Hanson, J., and H.E. Huxley. 1953. Structural basis of the cross-striations in muscle. Nature. 172:530–532. 10.1038/172530b0 13099257

[bib14] Haselgrove, J.C., and H.E. Huxley. 1973. X-ray evidence for radial cross-bridge movement and for the sliding filament model in actively contracting skeletal muscle. J. Mol. Biol. 77:549–568. 10.1016/0022-2836(73)90222-2 4541885

[bib15] Huxley, H.E., and W. Brown. 1967. The low-angle x-ray diagram of vertebrate striated muscle and its behaviour during contraction and rigor. J. Mol. Biol. 30:383–434. 10.1016/s0022-2836(67)80046-9 5586931

[bib16] Huxley, H.E., A. Stewart, H. Sosa, and T. Irving. 1994. X-ray diffraction measurements of the extensibility of actin and myosin filaments in contracting muscle. Biophys. J. 67:2411–2421. 10.1016/S0006-3495(94)80728-3 7696481 PMC1225626

[bib17] Jiratrakanvong, J., J. Shao, M. Menendez, X. Li, J. Li, W. Ma, G. Agam, and T. Irving. 2018. MuscleX: software suite for diffraction X-ray imaging V1.13.1. BioCAT. 10.5281/zenodo.1195050

[bib18] Kawas, R.F., R.L. Anderson, S.R.B. Ingle, Y. Song, A.S. Sran, and H.M. Rodriguez. 2017. A small-molecule modulator of cardiac myosin acts on multiple stages of the myosin chemomechanical cycle. J. Biol. Chem. 292:16571–16577. 10.1074/jbc.M117.776815 28808052 PMC5633120

[bib19] Kiss, B., E.J. Lee, W. Ma, F.W. Li, P. Tonino, S.M. Mijailovich, T.C. Irving, and H.L. Granzier. 2018. Nebulin stiffens the thin filament and augments cross-bridge interaction in skeletal muscle. Proc. Natl. Acad. Sci. USA. 115:10369–10374. 10.1073/pnas.1804726115 30249654 PMC6187167

[bib20] Labeit, D., K. Watanabe, C. Witt, H. Fujita, Y. Wu, S. Lahmers, T. Funck, S. Labeit, and H. Granzier. 2003. Calcium-dependent molecular spring elements in the giant protein titin. Proc. Natl. Acad. Sci. USA. 100:13716–13721. 10.1073/pnas.2235652100 14593205 PMC263879

[bib21] Lehman, W., R. Craig, and P. Vibert. 1994. Ca^2+^ induced tropomyosin movement in *Limulus* thin filaments revealed by three-dimensional reconstruction. Nature. 368:65–67. 10.1038/368065a0 8107884

[bib22] Linari, M., E. Brunello, M. Reconditi, L. Fusi, M. Caremani, T. Narayanan, G. Piazzesi, V. Lombardi, and M. Irving. 2015. Force generation by skeletal muscle is controlled by mechanosensing in myosin filaments. Nature. 528:276–279. 10.1038/nature15727 26560032

[bib23] Ma, W., M. Childers, J. Murray, F. Moussavi-Harami, H. Gong, R. Weiss, V. Daggett, T. Irving, and M. Regnier. 2020. Myosin dynamics during relaxation in mouse soleus muscle and modulation by 2'-deoxy-ATP. J. Physiol. 598:5165–5182. 10.1113/JP280402 32818298 PMC7719615

[bib24] Ma, W., H. Gong, and T. Irving. 2018a. Myosin head configurations in resting and contracting murine skeletal muscle. Int. J. Mol. Sci. 19:2643. 10.3390/ijms19092643 PMC6165214 30200618

[bib25] Ma, W., H. Gong, V. Jani, K.H. Lee, M. Landim-Vieira, M. Papadaki, J.R. Pinto, M.I. Aslam, A. Cammarato, and T. Irving. 2022. Myofibril orientation as a metric for characterizing heart disease. Biophys. J. 121:565–574. 10.1016/j.bpj.2022.01.009 35032456 PMC8874025

[bib26] Ma, W., H. Gong, B. Kiss, E.J. Lee, H. Granzier, and T. Irving. 2018b. Thick-filament extensibility in intact skeletal muscle. Biophys. J. 115:1580–1588. 10.1016/j.bpj.2018.08.038 30266320 PMC6196444

[bib27] Ma, W., H. Gong, B. Kiss, E.J. Lee, H. Granzier, and T. Irving. 2019. Response to: Thick filament length changes in muscle have both elastic and structural components. Biophys. J. 116:985–986. 10.1016/j.bpj.2019.02.010 30850114 PMC6428942

[bib28] Ma, W., M. Henze, R.L. Anderson, H. Gong, F.L. Wong, C.L. Del Rio, and T. Irving. 2021. The super-relaxed state and length dependent activation in porcine myocardium. Circ. Res. 129:617–630. 10.1161/CIRCRESAHA.120.318647 34365814 PMC8416939

[bib29] Ma, W., and T.C. Irving. 2022. Small angle X-ray diffraction as a tool for structural characterization of muscle disease. Int. J. Mol. Sci. 23:3052. 10.3390/ijms23063052 35328477 PMC8949570

[bib30] Margossian, S.S., and S. Lowey. 1982. Preparation of myosin and its subfragments from rabbit skeletal muscle. Methods Enzymol. 85 Pt B:55–71. 10.1016/0076-6879(82)85009-x 6214692

[bib31] Markandran, K., J.W. Poh, M.A. Ferenczi, and C. Cheung. 2021. Regulatory light chains in cardiac development and disease. Int. J. Mol. Sci. 22:4351. 10.3390/ijms22094351 33919432 PMC8122660

[bib32] Matsubara, I. 1980. X-ray diffraction studies of the heart. Annu. Rev. Biophys. Bioeng. 9:81–105. 10.1146/annurev.bb.09.060180.000501 6994598

[bib33] Metzger, J.M., and R.L. Moss. 1992. Myosin light chain 2 modulates calcium-sensitive cross-bridge transitions in vertebrate skeletal muscle. Biophys. J. 63:460–468. 10.1016/S0006-3495(92)81614-4 1420891 PMC1262169

[bib34] Mijailovich, S.M., M. Prodanovic, C. Poggesi, M.A. Geeves, and M. Regnier. 2021. Multiscale modeling of twitch contractions in cardiac trabeculae. J. Gen. Physiol. 153:e202012604. 10.1085/jgp.202012604 33512405 PMC7852458

[bib35] Morimoto, K., and W.F. Harrington. 1974. Evidence for structural changes in vertebrate thick filaments induced by calcium. J. Mol. Biol. 88:693–709. 10.1016/0022-2836(74)90417-3 4449125

[bib36] Nag, S., and D.V. Trivedi. 2021. To lie or not to lie: Super-relaxing with myosins. Elife. 10:e63703. 10.7554/eLife.63703 33565963 PMC7875563

[bib37] Nag, S., D.V. Trivedi, S.S. Sarkar, A.S. Adhikari, M.S. Sunitha, S. Sutton, K.M. Ruppel, and J.A. Spudich. 2017. The myosin mesa and the basis of hypercontractility caused by hypertrophic cardiomyopathy mutations. Nat. Struct. Mol. Biol. 24:525–533. 10.1038/nsmb.3408 28481356 PMC5737966

[bib38] Padron, R., W. Ma, S. Duno-Miranda, N. Koubassova, K.H. Lee, A. Pinto, L. Alamo, P. Bolanos, A. Tsaturyan, T. Irving, and R. Craig. 2020. The myosin interacting-heads motif present in live tarantula muscle explains tetanic and posttetanic phosphorylation mechanisms. Proc. Natl. Acad. Sci. USA. 117:11865–11874. 10.1073/pnas.1921312117 32444484 PMC7275770

[bib39] Park-Holohan, S.J., E. Brunello, T. Kampourakis, M. Rees, M. Irving, and L. Fusi. 2021. Stress-dependent activation of myosin in the heart requires thin filament activation and thick filament mechanosensing. Proc. Natl. Acad. Sci. USA. 118:e2023706118. 10.1073/pnas.2023706118 33850019 PMC8072254

[bib40] Podlubnaya, Z.A., S.L. Malyshev, K. Nieznanski, and D. Stepkowski. 2000. Order-disorder structural transitions in synthetic filaments of fast and slow skeletal muscle myosins under relaxing and activating conditions. Acta Biochim. Pol. 47:1007–1017. 10.18388/abp.2000_3954 11996091

[bib41] Reconditi, M. 2006. Recent improvements in small angle X-ray diffraction for the study of muscle physiology. Rep. Prog. Physics. 69:2709–2759. 10.1088/0034-4885/69/10/R01 PMC2783642 19946470

[bib42] Reconditi, M., M. Caremani, F. Pinzauti, J.D. Powers, T. Narayanan, G.J. Stienen, M. Linari, V. Lombardi, and G. Piazzesi. 2017. Myosin filament activation in the heart is tuned to the mechanical task. Proc. Natl. Acad. Sci. USA. 114:3240–3245. 10.1073/pnas.1619484114 28265101 PMC5373356

[bib43] Reconditi, M., L. Fusi, M. Caremani, E. Brunello, M. Linari, G. Piazzesi, V. Lombardi, and M. Irving. 2019. Thick filament length changes in muscle have both elastic and structural components. Biophys. J. 116:983–984. 10.1016/j.bpj.2019.02.009 30837077 PMC6428937

[bib44] Risi, C.M., I. Pepper, B. Belknap, M. Landim-Vieira, H.D. White, K. Dryden, J.R. Pinto, P.B. Chase, and V.E. Galkin. 2021. The structure of the native cardiac thin filament at systolic Ca(2+) levels. Proc. Natl. Acad. Sci. USA. 118:e2024288118. 10.1073/pnas.2024288118 33753506 PMC8020778

[bib45] Sankaranarayanan, R., K. Kistamas, D.J. Greensmith, L.A. Venetucci, and D.A. Eisner. 2017. Systolic [Ca(2+) ]i regulates diastolic levels in rat ventricular myocytes. J. Physiol. 595:5545–5555. 10.1113/JP274366 28617952 PMC5556151

[bib46] Spudich, J.A. 2019. Three perspectives on the molecular basis of hypercontractility caused by hypertrophic cardiomyopathy mutations. Pflugers Archiv. 471:701–717. 10.1007/s00424-019-02259-2 30767072 PMC6475635

[bib47] Spudich, J.A., and S. Watt. 1971. The regulation of rabbit skeletal muscle contraction. I. Biochemical studies of the interaction of the tropomyosin-troponin complex with actin and the proteolytic fragments of myosin. J. Biol. Chem. 246:4866–4871. 10.1016/s0021-9258(18)62016-2 4254541

[bib48] Stewart, M.A., K. Franks-Skiba, S. Chen, and R. Cooke. 2010. Myosin ATP turnover rate is a mechanism involved in thermogenesis in resting skeletal muscle fibers. Proc. Natl. Acad. Sci. USA. 107:430–435. 10.1073/pnas.0909468107 19966283 PMC2806748

[bib49] Stull, J.T., K.E. Kamm, and R. Vandenboom. 2011. Myosin light chain kinase and the role of myosin light chain phosphorylation in skeletal muscle. Arch. Biochem. Biophys. 510:120–128. 10.1016/j.abb.2011.01.017 21284933 PMC3101293

[bib50] Szent-Gyorgyi, A.G. 2007. Regulation by myosin: How calcium regulates some myosins, past and present. Adv. Exp. Med. Biol. 592:253–264. 10.1007/978-4-431-38453-3_21 17278370

[bib51] Tobacman, L.S. 1996. Thin filament-mediated regulation of cardiac contraction. Annu. Rev. Physiol. 58:447–481. 10.1146/annurev.ph.58.030196.002311 8815803

[bib52] Walker, J.S., X. Li, and P.M. Buttrick. 2010. Analysing force-pCa curves. J. Muscle Res. Cell Motil. 31:59–69. 10.1007/s10974-010-9208-7 20490629 PMC2943343

[bib53] Walsh, M.P. 1994. Calmodulin and the regulation of smooth muscle contraction. Mol. Cell. Biochem. 135:21–41. 10.1007/BF00925958 7816054

[bib54] Yuan, C.C., K. Kazmierczak, J. Liang, W. Ma, T.C. Irving, and D. Szczesna-Cordary. 2022. Molecular basis of force-pCa relation in MYL2 cardiomyopathy mice: Role of the super-relaxed state of myosin. Proc. Natl. Acad. Sci. USA. 119:e2110328119. 10.1073/pnas.2110328119 35177471 PMC8872785

