# Supplementary material for: Cardiac myosin filaments are directly regulated by calcium
Source: J Gen Physiol. 2022 Nov 1;154(12):e202213213. doi: 10.1085/jgp.202213213 (PMC9629851; doi:10.1085/jgp.202213213)
Supplement: Table S1 — include parameters obtained from MYK-7660 characterization [file JGP_202213213_TableS1.docx]

**Table S1: Parameters obtained from MYK-7660 characterization.**

|  | Inhibitor (MYK-7660) | |
| --- | --- | --- |
|  | IC_50_ (95% CI) | Hill Slope (95% CI) |
| ATPase Actin-S1 ^Fig 1a^ | >100 μM | - |
| ATPase RTF-S1 ^Fig 1a^ | 7.8 (6.6 to 9.4) | 1.2 (1.0 to 1.5) |
| Ca^2+^ release rate ^Fig 1b^ | 3.0 (2.5 to 3.5) | 0.6 (0.5 10 1.0) |
| Force ^Fig. 1c^ | 3.1 (2.7 to 3.5) | 1.6 (1.4 to 1.9) |
