# Supplementary material for: Cardiac myosin filaments are directly regulated by calcium
Source: J Gen Physiol. 2022 Nov 1;154(12):e202213213. doi: 10.1085/jgp.202213213 (PMC9629851; doi:10.1085/jgp.202213213)
Supplement: Table S2 — include parameters obtained from fitting X-ray datasets to modified Hill equation [file JGP_202213213_TableS2.docx]

**Table S2: Parameters obtained from fitting x-ray datasets to modified Hill equation.**

|  | Control | | Inhibitor (MYK-7660) | |
| --- | --- | --- | --- | --- |
|  | pCa_50_ (95% CI) | Hill Slope (95% CI) | pCa_50_ (95% CI) | Hill Slope (95% CI) |
| Δ I_1,1_/I_1,0_  ^Fig 2b^ | 5.7 (5.5 to 5.9) | 0.8 (0.3 to 1.4) | 5.6 (3.5 to 5.9) | 1.5 (0.9 to 2.9) |
| I_MLL1_ ^Fig. 3a^ | 6.1 (6.0 to 6.2) | 1.5 (1.1 to 2.0) | 6.1 (5.9 to 6.2) | 1.4 (0. 9 to 2.2) |
| I_M3_ ^Fig. 3b^ | 6.4 (6.2 to 6.5) | 1.1 (0.8 to 1.4) | 6.2 (6.0 to 6.3) | 1.3 (0.8 to 2.0) |
| I_M6_ ^Fig. 3c^ | 6.0 (5.8 to 6.1) | 1.0 (0.6 to 1.4) | 6.0 (5.8 to 6.1) | 1.0 (0.6 to 1.4) |
| S_M6_ ^Fig. 3d^ | 6.3 (6.1 to 6.6) | 1.4 (0.7 to 2.3) | 6.2 (6.0 to 6.4) | 1.4 (0.7 to 2.6) |
| Force ^Fig. 1d^ | 5.9 (5.8 to 6.0) | 1.3 (1.1 to 1.6) | N/A | N/A |
